# Audiovisual Biofeedback-Based Trunk Stabilization Training Using a Pressure Biofeedback System in Stroke Patients: A Randomized, Single-Blinded Study

**DOI:** 10.1155/2017/6190593

**Published:** 2017-12-20

**Authors:** Sangwoo Jung, Kyeongjin Lee, Myungjoon Kim, Changho Song

**Affiliations:** ^1^Department of Physical Therapy, Gimcheon University, Gimcheon, Republic of Korea; ^2^Department of Physical Therapy, Kyungdong University, Gangwon, Republic of Korea; ^3^Department of Physical Therapy, Sahmyook University, Seoul, Republic of Korea

## Abstract

The purpose of this study was to assess the effects of audiovisual biofeedback-based trunk stabilization training using a pressure biofeedback system (PBS) in stroke patients. Forty-three chronic stroke patients, who had experienced a stroke more than 6 months ago and were able to sit and walk independently, participated in this study. The subjects were randomly allocated to an experimental group (*n* = 21) or a control group (*n* = 22). The experimental group participated in audiovisual biofeedback-based trunk stabilization training for 50 minutes/day, 5 days/week, for 6 weeks. The control group underwent trunk stabilization training without any biofeedback. The primary outcome of this study was the thickness of the trunk muscles. The secondary outcomes included static sitting balance ability and dynamic sitting balance ability. The thickness of the trunk muscles, static sitting balance ability, and dynamic sitting balance ability were significantly improved in the experimental group compared to the control group (*p* < 0.05). The present study showed that trunk stabilization training using a PBS had a positive effect on the contracted ratio of trunk muscles and balance ability. By providing audiovisual feedback, the PBS enables accurate and effective training of the trunk muscles, and it is an effective method for trunk stabilization.

## 1. Introduction

In stroke patients, the normal muscle stiffness is lost, muscle strength is impaired, and postural control becomes difficult due to asymmetry [1]. Balance disabilities lead to an increased risk of falls and also affect activities of daily living (ADL) [2, 3]. Therefore, improving balance is one of the major goals of rehabilitation in patients with stroke-induced hemiplegia. Although numerous studies have been conducted on improvement in balance [4], a large number of stroke patients continue to have difficulties in these areas.

Postural balance involves control of individual components of the musculoskeletal system, which is achieved by cerebellar integration of information from the vestibular organs and the visual and proprioceptive information [5]. Of these, impaired proprioception and lack of appropriate control of muscle contraction, which are sequelae of brain damage, are the primary concerns in stroke patients [6]. The limb asymmetry makes it difficult for the patient to achieve trunk control [7, 8]. Instability and impaired trunk control lead to problems in sitting balance [2, 9]. Therefore, in order to maintain postural balance, trunk control and stabilization need to be prioritized [10, 11]. Trunk control helps maintain balance by regulating the shifting of body weight during postural changes on various surfaces [9]. Stabilizing the body proximally is important for efficient movement of the limbs [7].

When aiming to improve postural balance clinically, the focus has been on pelvic movements and trunk stability. Trunk stability training helps to control trunk movements by synergistically activating the postural muscles, namely, the abdominal and multifidus muscles, through pelvic and abdominal training [12, 13]. Additionally, previous research has shown that strengthening the transversus abdominis (TrA) provides stability to the sacroiliac joints and is therefore important for improving trunk stability [14]. However, during such training, the subjects demonstrated compensatory movement patterns using muscles other than the target muscles. Moreover, difficulty in recognizing the use of trunk muscles during training was another factor that interfered with trunk training [15, 16].

The trunk muscles are divided into deep muscles and global muscles. The TrA and internal oblique (IO) are deep muscles that contribute to trunk stabilization, while the external oblique (EO) and rectus abdominis (RA) are global muscles that contribute to dynamic movements [12, 13]. Recently, several studies have reported that TrA training is effective for trunk stabilization, and training for facilitating isolated TrA contraction has been reported in low back pain patients [17, 18]. The abdominal drawing-in maneuver (ADIM) is often used for this purpose [19]. Real-time ultrasound imaging (RTUI) is used during training for a more precise recognition of muscle contraction techniques [20].

Several studies have recently attempted trunk stabilization training in stroke patients, which includes trunk control training through proprioceptive exercise and tasks, weight-shift training, and visual and auditory feedback training [21–24]. However, although the majority of these studies found that trunk stabilization training affected trunk performance, the effects are still not clear, and there have been no specific reports on the effect of trunk stabilization training on trunk muscles in stroke patients.

Recently, an exercise method that utilizes a pressure biofeedback unit (PBU) was introduced; this method promotes symmetrical contraction of the trunk muscles to effectively train the patient for isolated TrA contraction [25]. A PBU involves placing an air pocket between the patient's lower back and a hard surface and using a pressure meter; the extent of movement is verified in real time. This method is used frequently in stabilization of the back or neck. The feedback from the PBU has been shown to be effective in improving trunk stability in low back pain patients by promoting recognition of the correct contraction techniques [26].

Hence, the present study aimed to verify these effects in stroke patients by educating them in the precise exercise methods for isolated TrA contraction using RTUI and applying audiovisual biofeedback-based trunk stabilization training using a PBS.

## 2. Subjects and Methods

### 2.1. Subjects

 The subjects in this study were all individuals diagnosed with stroke and admitted to “D” rehabilitation hospital as inpatients in South Korea. The inclusion criteria were as follows: hemiplegic patients who had been diagnosed with stroke at least 6 months ago; patients who had experienced only 1 stroke; patients who scored at least 24 points on the Mini-Mental State Examination; patients capable of unassisted sitting for at least 10 minutes; patients capable of gait for a distance of at least 10 m independently, with or without assistive tools; and patients with a Brunnstrom motor recovery stage of at least 4. The exclusion criteria were as follows: patients participating in another experiment that could affect this study; patients with visual or auditory abnormalities such as vestibular disease, cerebellar disease, unilateral neglect, or apraxia; patients with brain abnormalities outside of the stroke region such as the cerebellum or brainstem; patients with a surgical condition such as a lower limb fracture or peripheral nerve damage; patients with severe renal, musculoskeletal, or cardiovascular disease that would impair training; and patients with visual disability, loss of visual field, or auditory disability. Prior to the study, the aims and procedures of the study were explained to all participants, who signed the research participation consent form of their own free will. The entire study procedure was approved in advance by the Institutional Review Board of the University of Sahmyook.

### 2.2. Sample Size

This study used a randomized, single-blinded design. To determine the sample size, the G-Power 3.19 software was used [27]. To calculate the sample size, the probability of alpha error and power were set at 0.05 and 0.8, respectively. In addition, the effect size was set at 0.92, based on the trunk ability results in a prior pilot test. Therefore, a sample size of 20 patients per group was necessary. By estimating a dropout rate of about 15%, 23 participants per group needed to be recruited for randomization.

### 2.3. Procedure

**Figure 1:**
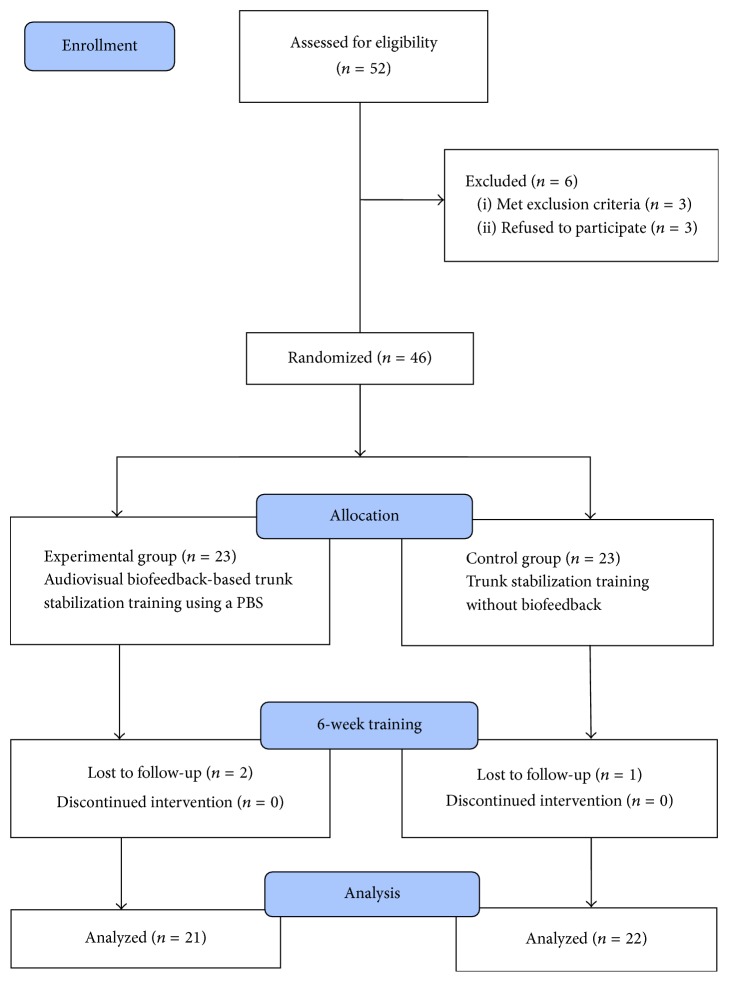
Flow diagram based on CONSORT.

Among 52 hospitalized stroke patients, 46 patients met the inclusion criteria, and these were randomly allocated to an experimental group or a control group of 23 patients each. Random allocation software was used to minimize selection bias [28]. The experimental group used a PBU and performed audiovisual biofeedback-based trunk stabilization training for 50 minutes/session, 5 sessions/week, for 6 weeks. The control group performed identical trunk stabilization training, but without the PBU. The changes in thickness of trunk muscle, static sitting balance ability, and dynamic sitting balance ability were assessed before and after the training. The tests were performed by the trained assessors, and the assessors were blinded to the subjects' groups. The subjects who became unable to participate in the program during the study due to a change in medical status, or who were unable to receive the posttraining tests, were excluded from the final analysis. In the experimental group, statistical analysis was conducted on 21 patients, excluding 2 who were unable to participate in posttraining tests, and in the control group, the final analysis was conducted on 22 patients, excluding 1 patient who was unable to participate in posttraining tests (Figure 1).

#### 2.3.1. ADIM Education

Prior to the training, all subjects in both groups underwent ADIM education. The education was provided by a skilled assessor. RTUI was used to educate the subjects in isolated TrA contraction, without contraction of the EO. With the patient in the supine hook-lying position, ultrasound gel was applied to the region of measurement, and the middle of the probe was placed 2.5 cm anterior to the mid-axillary line, at the midpoint between the 12th rib and the iliac crest. During the measurement, the patient was instructed to slowly and gently pull the lower abdomen below the navel in. The patient was instructed not to move the upper abdomen, back, or pelvis and to focus on the monitor during the movement. The patients were educated until they were capable of performing an isolated TrA contraction [29].

#### 2.3.2. Audiovisual Biofeedback-Based Trunk Stabilization Training with a PBS

The patients assumed the supine position with the knees raised (supine hook-lying position). A pillow was used to maintain a neutral cervical spine, and the patient was instructed to release the tension in the neck, which was checked by the sternocleidomastoid muscles. Three PBSs (Achievo CST, V2U Healthcare, Pte., Ltd., Singapore) were used to provide audiovisual biofeedback-based trunk stabilization training. Consisting of an inflatable cushion, a computer system, and a monitor, it detects pressure changes, and when the pressure falls out of a certain range, a red light appears on the monitor, and a warning sound is heard.

The monitor was placed in the direction of the patient's gaze, so that the patient could look comfortably at the monitor during the exercise. A stabilizer was placed below the anterior curvature of the low back, and the lower part of the stabilizer was aligned with the posterior superior iliac spine. Once the patient adopted the correct posture for the exercise, the pressure of the stabilizer was set to 40 mmHg, and the exercise range was selected. The acceptable pressure range started at 20% and decreased by 5% for each stage. The stabilizer pressure was maintained at 40 mmHg, so that the patient would perform the ADIM [29, 30]. If the patient was unable to maintain the proper ADIM, and the pressure exceeded the acceptable range, a red light was seen on the monitor and a warning sound was heard.

**Figure 2:**
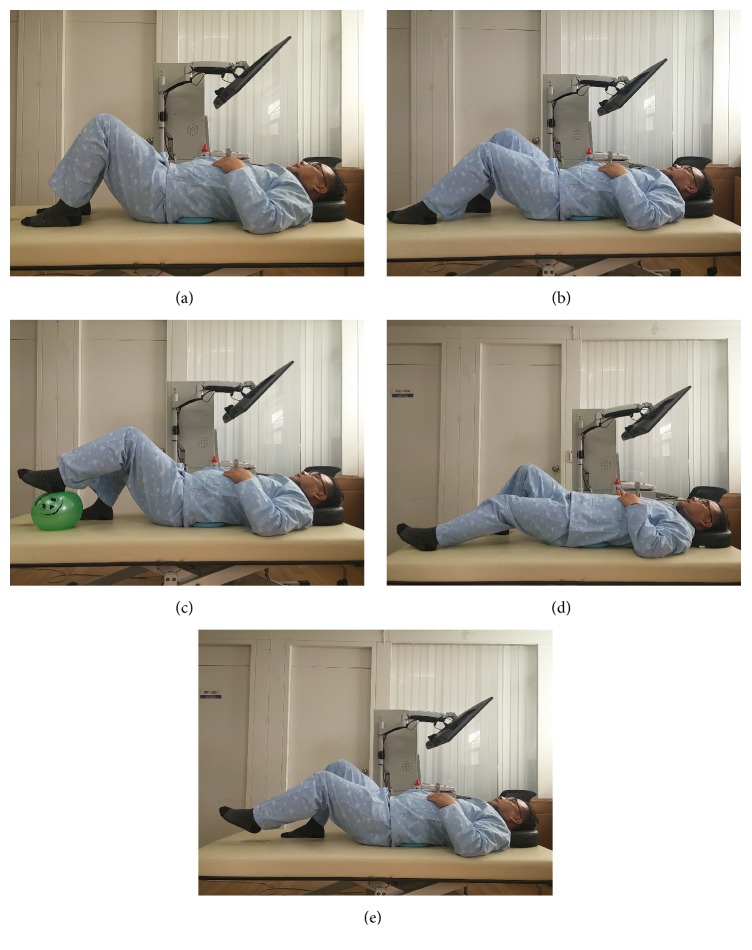
Audiovisual biofeedback-based trunk stabilization training using a pressure biofeedback system. (a) Starting position, (b) semisliding, (c) ball sliding, (d) sliding, and (e) raised sliding.

To stabilize the trunk, 4 stages of the sliding movement were performed, with the stabilizer pressure maintained. During the sliding exercise, the patient fully extends the bent knees and then returns to the original position. The first stage is semisliding, where the feet remain on the ground, and the patient only performs the exercise through half the total range. The second stage is ball sliding, where the full range of sliding is performed, but the patient's feet are placed on top of a ball to make the action easier. The third stage is sliding, where the patient's feet remain on the ground, and the patient fully extends the knees before returning to the original position. The fourth stage is raised sliding, where sliding is performed with the feet lifted slightly off the ground [31–33] (Figure 2). Each movement was performed as a set of 10 repetitions [34]. The movements were performed gradually according to individual ability, with the patients advancing to the next stage when they achieved a success rate of at least 90%. Even those who were able to perform the latter stages of the exercise had to start with the first stage of the exercise. Patients were instructed to breathe normally during the exercise, which was monitored, and in the event of breathing difficulties, the patient was allowed to rest before resuming the exercise. The therapist provided assistance to those who required support on the affected side. Care was taken to avoid unnecessary hypertonus in other areas during the exercise. The control group performed the same movements as described above but without any biofeedback.

### 2.4. Measurements

The outcomes were measured by assessors who were blinded to subjects' group placement before intervention and after completing the 6-week training. The primary outcome was thickness of the trunk muscles. Secondary outcome measures were used to estimate the clinical relevance of the primary outcome results. Static sitting balance ability and dynamic sitting balance ability were assessed for the subjects in each group.

Ultrasonography equipment (Achievo CST, V2U Healthcare, Pte., Ltd., Singapore) was used to measure the thickness of the trunk muscles. A 5 MHz convex transducer was used. With the patient in the supine hook-lying position, ultrasound gel was applied to the measurement area, and the transducer was placed 2.5 cm anterior to the mid-axillary line on the right side of the trunk, at the midpoint between the 12th rib and the iliac crest. Measurements were performed on the unaffected and affected sides during contraction and relaxation. To measure the thickness during contraction, the patient adopted the ADIM position, after the patient was educated on this position. The patient was instructed to pull their lower abdomen back towards the spine in the final 2/3 of the normal exhalation phase [29, 30]. Each measurement was repeated 3 times. On the ultrasound imaging screen, the thickness of the TrA, IO, and EO was measured by drawing a vertical line to a point 2.5 cm from the myofascial junction of the TrA and thoracolumbar fascia [35]. The average of the 3 measurements was used in the final analysis. This study compared symmetric and contracted ratio after measurement of thickness of trunk muscle. Symmetric ratio is calculated as unaffected side/affected side and the contracted ratio as contraction/rest.

To evaluate static sitting balance, the Good Balance System (GB300; Metitur Ltd., Finland) was used in this study. The system consists of an equilateral triangular force platform, which is connected to a computer using a 3-channel amplifier with an A/D converter. The sampling frequency used was 50 Hz. This equipment is used to assess balance in patients with senile conditions as well as those with stroke and is widely available [36]. The Good Balance System measures the medial-lateral and anterior-posterior sway speed and velocity moment in the sitting position in stroke patients. The intrarater reliability of the Good Balance System was reported as intraclass correlation coefficients (*r*) of 0.51–0.74 (anterior-posterior speed) and 0.63–0.83 (right-left speed) [37]. To assess static balance, the patients sit on a high chair with the feet not contacting the floor. The patients were asked to look at a point (10 cm diameter) that was at a distance of 1 m in front of them for 30 s, while their balance was measured. This test was repeated 3 times. The same procedure was repeated with the patients' eyes closed. For the data analysis, the average values were recorded.

Dynamic balance in the sitting position was assessed using the modified functional reach test (MFRT). A stick ruler was set at the patient's acromial height and fixed on the wall, with the patient seated comfortably on a stool. The stick ruler was used to measure distance during the test. The patient's hips and knees were flexed to 90°, with the chair and popliteal area 5 cm apart and the feet in contact with the ground. For anterior measurements, the shoulder was flexed to 90° with the elbow fully extended, and the subject moved his or her upper extremities and trunk as forward as possible. The distance from the starting position to the ending position of the middle finger tip was measured using the stick ruler. For lateral measurements on the unaffected side, the shoulder was abducted to 90° with the elbow fully extended. The subject moved his or her upper extremities and trunk towards the unaffected side to the maximum range possible. The distance from the starting position to the ending position of the middle finger tip was measured using the stick ruler. All evaluations were repeated 3 times, and the average values were recorded. The interrater reliability of this test was reported as *r* = 0.97, indicating excellent reliability [38].

### 2.5. Data Analysis

Descriptive statistics were used to summarize baseline characteristics. The Shapiro-Wilk test was used to test the variables for normality. The Chi-square test was used for comparison of categorical dependent variables between the groups. The independent *t*-test was used for a comparison of change in thickness of trunk muscles and balance ability values between the experimental and control groups. Comparisons between pre-and posttreatment data within each group were analyzed using a paired *t*-test. SPSS version 19.0 for Windows was used to perform all analyses and *p* values < 0.05 were regarded as significant.

## 3. Results

General characteristics of 43 subjects with chronic stroke who fulfilled the inclusion criteria for the study are shown in Table 1. No significant differences in general characteristics and dependent variables were observed between the experimental and control group.

Results for the primary outcomes are shown in Table 2. Regarding changes in thickness of trunk muscles, the contracted ratio of the TrA in the experimental group was significantly increased after the intervention (*p* < 0.05). However, the control group displayed no significant difference. After training, the contracted ratios of the IO in both the experimental and control groups were significantly increased (*p* < 0.05). No significant improvement was observed in the experimental group compared with the control group. The contracted ratio of the EO in the control group was significantly increased (*p* < 0.05). However, the experimental group displayed no significant difference. In addition, after the 6-week training, the symmetric ratios of all muscles in both the experimental and control groups were not increased significantly.

Results for the secondary outcomes are shown in Table 3. Regarding changes in static sitting balance ability, medial-lateral sway speed, anterior-posterior sway speed, and velocity of moment in both the experimental and control groups regardless of their vision displayed significant improvement after the intervention. In addition, the improvement was significantly better in the experimental group than in the control group (*p* < 0.05).

In the MFRT, the reaching distances with the forward, affected side, and unaffected side movements in both the experimental and control groups were significantly increased after the intervention (*p* < 0.05). In addition, the training resulted in significantly larger improvement in all three variables in the experimental group than in the control group (*p* < 0.05).

## 4. Discussions

The effects of audiovisual trunk stabilization training in patients with neurological conditions such as low back pain and stroke have received a lot of attention, and several studies have been conducted on this topic. As most studies are focused on the trunk stabilizing effects of strengthening trunk muscles, there is still a lack of studies demonstrating the effects of trunk stabilization on functional activity [21–24].

Therefore, the primary aim of the present study was to verify the effects of 6 weeks of audiovisual biofeedback-based trunk stabilization training using a PBU on trunk muscles in stroke patients. The secondary aim of this study was to verify the carryover effect of the training on static sitting balance and dynamic sitting balance.

Proximal stability must be achieved prior to distal movement, whereas functional activity and activation of the trunk muscles are essential preconditions for spinal stabilization during exercise [7]. The trunk muscles are categorized into local and global muscles [39]. Global muscles are located near the surface and include the EO and RA [40]. These muscles provide strength for gross movements of the trunk and not only move the spine, but also enable shifting of loads between the chest and pelvis [12, 41]. The local muscles are located deeper and include the multifidus, TrA, and IO; these provide stability to the lumbosacral spine [13].

Karatas et al. [2] reported weakness of trunk muscles in stroke patients compared to elderly individuals without stroke. Dickstein et al. [15] evaluated the trunk muscles in stroke patients and elderly individuals using electromyography and reported that the trunk muscles in stroke patients showed delayed contraction on the affected side compared to the trunk muscles in elderly individuals and that symmetrical contraction of the trunk muscles was also significantly impaired in stroke patients. Moreover, according to the results of previous studies, while healthy adults show activation of TrA prior to movement, subjects with impaired trunk stability, such as those with low back pain patients, had delayed TrA activation, and trunk stabilization training to strengthen the multifidus and TrA was reported to contribute considerably to lumbar stabilization [18, 42, 43].

The aim of trunk stabilization training is to improve trunk stability by strengthening the deep muscles and promoting synergistic action [12, 13]. A large number of studies have used reeducation of muscle control and muscle performance to achieve trunk stabilization, and these studies describe 3 stages of segmental control [44]. In the first stage, feedback is provided to stimulate and activate the local muscles. Feedback methods include palpation, EMG, and RTUI, and these methods aim to increase use of the local muscles and suppress use of the global muscles. In the second stage, the aim is to improve motor control and movements using closed chain exercises. This stage involves gradual weight loading while maintaining co-contraction of the local muscles. The third stage uses open chain exercises and aims to train the patient to maintain local segmental control while performing functional activities.

From a biomechanical perspective, the present study aimed to stimulate and activate local muscles using RTUI and visual biofeedback and used a compound method combining closed chain and open chain exercises using sliding motion. In a study by Lee et al. [45], palpation feedback was used to investigate activation of local muscles. This method is frequently used for trunk stabilization training in clinical practice; however, selective contraction of the deep muscles without biofeedback seems to be difficult. Previously, studies have been conducted using RTUI or pressure feedback to overcome this difficulty. Pressure feedback was used in patients with lower back pain patients to facilitate independent contraction of TrA, and it was found to be effective for stabilization of the sacroiliac joint [7, 14]. RTUI has been reported to be more accurate and more effective than pressure feedback [44, 46, 47]; Seo et al. [48] therefore used RTUI in stroke patients to effectively implement trunk stabilization training. Using RTUI may be effective; however, it has the following disadvantages: It requires expensive equipment; patients experience some discomfort when the ultrasound transducer is placed against their skin; and patients have difficulty interpreting the ultrasound images. Therefore, the present study used a PBS to provide audiovisual feedback. It is thought that a PBU could be used easily in clinical practice.

In the present study, ultrasound was used to measure changes in the thickness of the trunk muscles following training, and trunk stabilization training was found to be effective. After training, the experimental group showed a significant improvement in the contraction ratio of the TrA, at 28% on the affected side and 11% on the unaffected side, and the IO, at 4% on the affected side and 6% on the unaffected side. Conversely, the control group showed a significant change in the contraction ratio of the IO, at 4% on the affected side and 7% on the unaffected side, and the EO, at 8% on the affected side and 11% on the unaffected side.

Vasseljen and Fladmark [20] applied the ADIM using RTUI in patients with lower back pain patients and reported an increase of 3% in the thickness of TrA. Seo et al. [48] applied trunk stabilization exercises using a PBU in chronic stroke patients and reported results similar to those of the present study, with an improvement of 17% and 15% in the thickness of TA during contraction on the affected and unaffected sides, respectively.

The trunk stabilization training and feedback used in the present study promoted isolated contraction of the deep muscle, TrA, and improved trunk stability with strengthening of the TrA. Compared to the control group, the experimental group subjects were thought to have achieved more effective learning of selective TrA contraction, because they were provided with audiovisual feedback. In addition, the effect of selective training with feedback combined with trunk stability training in the present study is thought to have activated the tonic stabilizing muscle, TrA, by facilitating co-contraction in a multidimensional manner. Hodges and Richardson [43] also reported that motor control, achieved by combining functional movements with PBS training, is more effective at promoting activation of local muscles.

In the experimental group, because subjects were given feedback to help maintain a neutral pelvic position during exercise, lumbopelvic motion was restricted, and the TrA could be activated more than the other abdominal muscles [49]. Meanwhile, because the control group underwent training without feedback, it was difficult to maintain the precise posture during exercise, and it is thought that these patients performed the actions with a posterior pelvic tilt. When the pelvis is tilted posteriorly, the global muscles such as the RA and EO are activated more than the muscles of the anterolateral abdomen, and this is considered to be an undesirable pattern for lumbar stabilization [50].

Although the symmetric ratio improved in both groups, there was no significant difference. This may be because although muscle activation improved on the affected side, it improved to the same extent or more on the unaffected side. Moreover, with the exercise methods used in the present study, it was not possible to selectively target the unaffected or affected side. Due to the anatomical nature of the trunk muscles, it is very difficult to perform the exercise only on one side. Therefore, improving the symmetric ratio with trunk stabilization training is expected to be difficult.

The present study was conducted under the assumption that changes in trunk muscles would affect static and dynamic sitting balance.

Stroke patients show a greater impairment of trunk proprioception with an increasing trunk reposition error [9], and improvement of proprioception in stroke patients is reported to positively affect trunk control [51]. Mudie et al. [51] applied body position awareness training in stroke patients and reported improved proprioception. Gruber and Gollhofer [52] used trunk control training on an unstable surface and found that it was very effective at increasing proprioceptive input to the neuromuscular system. Additionally, Kawato et al. [53] reported that trunk stabilization training improved postural control when correcting errors through feedback. The present study was also designed to utilize a PBS, because training with feedback was thought to improve trunk stability by providing awareness of the trunk position and improving postural control. Hence, improvement in trunk stability is thought to have affected the patient's sitting balance.

In the present study, both groups showed a significant improvement in static and dynamic sitting balance, with a greater effect in the experimental group than in the control group. Among the various factors that affect sitting balance in stroke patients, stabilization of the trunk muscles is very important. Previous studies have also shown that improving trunk stability improves sitting balance ability [21, 54]. The experimental group is thought to have shown improved sitting balance, because the TrA was strengthened using trunk stabilization training and feedback. The TrA provides trunk stability by acting preemptively in feed-forward postural control and various postural changes that increase the spinal load. Conversely, the control group showed improvements in the IO and EO without any feedback, and the global muscles in this group seem to have contributed to trunk stabilization by acting as stabilizers. Combined training of the TrA and EO can be predicted to be even more effective, although this cannot be demonstrated clearly in our results. This should be confirmed by future research.

Both groups also showed improvement on the MFRT, which tests not only static balance, but also reaching with the arms, while maintaining a seated position. The experimental group showed a significant improvement of 10% in the forward direction, 13% on the unaffected side, and 18% on the affected side on the MFRT, and this improvement was greater than that shown by the control group, which showed improvements of 4%, 6%, and 9%, respectively. Lee et al. [55] applied trunk stabilization training with visual feedback in chronic stroke patients, who showed a significant improvement on the MFRT; this is consistent with the results of our study.

When a patient attempts to maintain balance in the seated position, compensatory movements of the limbs can occur to control the anterior-posterior sway. Control of medial-lateral movements is closely related to trunk control [56]. The present study showed a significant improvement in the medial-lateral direction, demonstrating that the intervention in this study was closely related to trunk control.

In the present study, as both groups performed trunk stabilization exercise, it was not possible to be precise about the effects of stabilization training. In addition, the 6-week intervention duration was not long enough to produce changes in gait.

## Figures and Tables

**Table 1:** Characteristics of subjects in the experimental and control groups (*N* = 43).

Characteristics	Experimental group (*n* = 21)	Control group (*n* = 22)	*χ* ^2^/*t* (*p*)
Gender (male/female)	14/7	13/9	0.607 (0.264)
Affected side (right/left)	12/9	11/11	0.639 (0.220)
Stroke type (infarct/hemorrhage)	15/6	14/8	0.586 (0.297)
Age (year)	62.52 ± 8.82	64.55 ± 10.67	0.675 (0.503)
Height (cm)	165.29 ± 6.90	161.73 ± 9.47	1.403 (0.168)
Weight (kg)	62.67 ± 7.45	60.80 ± 9.30	0.725 (0.473)
BMI (point)	22.93 ± 2.35	23.14 ± 2.02	0.316 (0.754)
Duration of stroke (month)	15.38 ± 7.45	16.45 ± 6.96	0.488 (0.628)
MMSE (score)	25.81 ± 1.29	25.77 ± 0.92	0.108 (0.914)

*Note*. BMI: body mass index; MMSE: Mini-Mental State Examination. Values are expressed as mean ± standard deviation.

**Table 2:** Comparison of thicknesses of trunk muscles within group and between groups (*N* = 43).

Variables	Experimental group (*n* = 21)			Control group (*n* = 22)			Significance of change scores
	Baseline	Post	Change score	Baseline	Post	Change score	*t* (*p*)
Contracted ratio							
TrA-affected	0.96 ± 0.14	1.23 ± 0.09	0.26 ± 0.08^*∗*^	0.93 ± 0.09	0.96 ± 0.16	0.03 ± 0.19	5.260 (0.000)
TrA-unaffected	1.36 ± 0.05	1.51 ± 0.13	0.15 ± 0.13^*∗*^	1.35 ± 0.04	1.36 ± 0.15	0.01 ± 0.14	3.331 (0.002)
IO-affected	1.14 ± 0.02	1.19 ± 0.05	0.06 ± 0.05^*∗*^	1.15 ± 0.06	1.20 ± 0.11	0.05 ± 0.09	0.448 (0.656)
IO-unaffected	1.31 ± 0.16	1.39 ± 0.18	0.08 ± 0.09^*∗*^	1.27 ± 0.16	1.36 ± 0.15	0.09 ± 0.19	0.338 (0.737)
EO-affected	1.38 ± 0.09	1.40 ± 0.15	0.02 ± 0.08	1.35 ± 0.07	1.46 ± 0.10	0.11 ± 0.11^*∗*^	2.938 (0.005)
EO-unaffected	1.47 ± 0.16	1.45 ± 0.14	−0.02 ± 0.15	1.41 ± 0.16	1.56 ± 0.19	0.16 ± 0.17^*∗*^	3.551 (0.001)
Symmetric ratio							
TrA-rest	1.16 ± 0.11	1.15 ± 0.10	−0.01 ± 0.04	1.14 ± 0.11	1.15 ± 0.11	0.01 ± 0.04	1.808 (0.400)
TrA-contract	1.23 ± 0.12	1.16 ± 0.18	−0.07 ± 0.19	1.24 ± 0.15	1.22 ± 0.17	−0.02 ± 0.04	1.346 (0.186)
IO-rest	1.14 ± 0.10	1.12 ± 0.06	−0.02 ± 0.06	1.15 ± 0.10	1.14 ± 0.10	−0.01 ± 0.08	0.641 (0.525)
IO-contract	1.26 ± 0.10	1.25 ± 0.07	−0.01 ± 0.08	1.26 ± 0.10	1.26 ± 0.10	−0.01 ± 0.09	0.329 (0.744)
EO-rest	1.19 ± 0.12	1.20 ± 0.11	0.01 ± 0.07	1.21 ± 0.13	1.21 ± 0.12	0.00 ± 0.07	0.320 (0.751)
EO-contract	1.32 ± 0.12	1.31 ± 0.10	−0.01 ± 0.03	1.34 ± 0.14	1.31 ± 0.15	−0.03 ± 0.14	0.537 (0.594)

*Note*. Values are expressed as mean ± standard deviation; TrA: transverse abdominal muscle; IO: internal oblique muscle; EO: external oblique muscle. ^*∗*^Significant difference within group.

**Table 3:** Comparison of secondary measures within group and between groups (*N* = 43).

Variables	Experimental group (*n* = 21)			Control group (*n* = 22)			Significance of change scores
	Baseline	Post	Change score	Baseline	Post	Change score	*t* (*p*)
Static sitting balance ability							
EO-MLS (mm/s)	3.99 ± 1.28	2.99 ± 0.82	−1.00 ± 0.88^*∗*^	3.76 ± 1.19	3.33 ± 1.01	−0.43 ± 0.91^*∗*^	2.114 (0.041)
EO-APS (mm/s)	5.73 ± 1.40	4.64 ± 1.38	−1.09 ± 0.74^*∗*^	5.83 ± 1.19	5.26 ± 1.30	−0.57 ± 0.86^*∗*^	2.148 (0.038)
EO-VM (mm/s^2^)	4.07 ± 1.35	2.74 ± 1.33	−1.33 ± 0.78^*∗*^	4.01 ± 1.11	3.27 ± 1.26	−0.74 ± 1.04^*∗*^	2.088 (0.043)
EC-MLS (mm/s)	4.18 ± 1.37	3.17 ± 0.99	−1.01 ± 0.74^*∗*^	4.48 ± 1.16	3.98 ± 1.36	−0.50 ± 0.73^*∗*^	2.256 (0.029)
EC-APS (mm/s)	5.90 ± 1.69	4.86 ± 1.42	−1.04 ± 0.71^*∗*^	5.96 ± 1.01	5.43 ± 1.00	−0.53 ± 0.64^*∗*^	2.479 (0.017)
EC-VM (mm/s^2^)	4.87 ± 2.10	3.64 ± 1.67	−1.23 ± 0.89^*∗*^	4.94 ± 1.89	4.35 ± 1.81	−0.59 ± 1.06^*∗*^	2.143 (0.038)
Dynamic sitting balance ability (cm)							
MFRT-forward	304.21 ± 110.65	333.82 ± 101.19	29.61 ± 24.66^*∗*^	281.21 ± 123.43	291.45 ± 123.36	10.23 ± 8.59^*∗*^	3.474 (0.001)
MFRT-unaffected	174.10 ± 49.24	196.47 ± 55.93	22.36 ± 20.80^*∗*^	156.74 ± 61.00	166.31 ± 61.99	9.57 ± 9.12^*∗*^	2.632 (0.012)
MFRT-affected	93.63 ± 36.56	110.90 ± 40.43	17.27 ± 14.21^*∗*^	83.08 ± 37.02	90.49 ± 38.09	7.41 ± 6.37^*∗*^	2.960 (0.005)

*Note*. Values are expressed as mean ± standard deviation; EO: eye opened; EC: eye closed; MLS: medial-lateral speed; APS: anterior-posterior speed; VM: velocity moment; MFRT: modified functional reach test. ^*∗*^Significant difference within group.
